# Impact of COVID-19 on mental health in China: analysis based on sentiment knowledge enhanced pre-training and XGBoost algorithm

**DOI:** 10.3389/fpubh.2023.1170838

**Published:** 2023-07-07

**Authors:** Ru Huang, Xiuli Wang

**Affiliations:** School of Economics and Management, Nanjing University of Science and Technology, Nanjing, China

**Keywords:** environmental health, one health, COVID-19, global health, China

## Abstract

Coronavirus disease 2019 (COVID-19) is causing a serious impact on the people living in countries across the entire world. The spread of this pandemic globally has led people worry every day about losing their jobs or even being threatened by the virus. This pandemic caused people to experience more serious psychological problems than we realized. However, there has been little research on how COVID-19 affects the mental health of the people. In this article, we attempted to use the social text data about COVID-19 on Sina Weibo (the largest “tweet” platform in China, and we will also call Weibo as tweet in the following content), to explore the impact of COVID-19 on the mental health of Chinese people. First, we fifilter the tweet data by selecting examples that contain COVID-19 and COVID-19 correlated keywords. However, we segment the filtered tweets, extract meaningful words, and construct a word vector sparse matrix as the measurement of every tweet. Then, for the model's labels, we use sentiment knowledge enhanced pre-training model (SKEP), a deep learning framework published by Baidu that measures the user's mental state. Through SKEP, we can obtain the probabilities of the user's positive and negative mental states. Finally, we use the XGBoost algorithm to study the relationship between the word vector sparse matrix and the mental health state of users. Our research shows that social text data can, indeed, reflect the mental health state of users to a large extent, and social data can be used to explore the impact of COVID-19 on mental health, which can help frame the public health policy.

## 1. Introduction

The COVID-19 caused by the Coronavirus strain SARS-CoV-2 is currently an epidemic (1). The World Health Organization (WHO) declared that the COVID-19 outbreak has become a public health emergency of international concern (2). The epidemic not only has a direct impact on the physical health of millions of people, but also has a huge influence on the mental health of people (3–5). The epidemic and lockdown will inevitably affect everyone's mental health, no matter how well the outbreak is contained.

A report released by the WHO in March 2022 shows that the COVID-19 epidemic has increased the mental pressure of people everywhere during the global pandemic. In 2020, the first year of pandemic, there was a significant 25% increase in the global prevalence of anxiety and depression. Moreover, young people were particularly affected. In addition, women were more severely affected than men, and people with underlying diseases were more likely to have mental health problems. One of the main reasons that can explain the widespread increase in mental health crisis across the globe is the fact that the social isolation brought about by the pandemic caused an unprecedented pressure, by limiting people's ability to work, seeking support from relatives, and restriction in participating in community activities. According to the WHO, although 90% of countries have included mental health and psychosocial support in their COVID-19 response plans, huge gaps and concerns are still prevalent among different countries (6). Since the COVID-19 epidemic started 2 years ago, people in many countries have experienced more mental health problems. However, mental health services also had to face serious challenges and disruptions, leaving a huge unmet need to bestow care and support to the most vulnerable people.

During most of the COVID-19 pandemic, mental health services were the most severely disrupted of all the basic health services. Many countries also reported that some life-saving mental health services, including suicide prevention, were seriously disrupted.

In Japan, one of the countries with the most detailed statistics and records of suicides in the world, the number of suicides in 2020 rose for the first time after 10 consecutive years of decline (7). Dr. Jia Wenting, a senior brain neurosurgeon and head of the Fangyuan Zaizai Clinic in Japan, said that the patients she met had various sources of psychological stress. These sources of stress included vaccine hesitancy, COVID-19 infection and isolation, loss of work and income, and family concerns.

Japan is not a special case. According to a survey conducted by the Ministry of Health of Singapore, in the first year of COVID-19 pandemic, 8.7% of the Singaporeans who were interviewed met the criteria of clinical depression, 9.4% met the criteria of anxiety, and 9.3% met the criteria of mild to severe stress (8). The main sources of psychological stress are the risk of COVID-19 infection among family members or friends, economic loss, and unemployment.

The cited examples illustrate the impact of the epidemic on mental health, and to study this influence even further, Gao et al. (9) and Hao et al. (10) collected information through questionnaires and surveys. Moreover, traditional methods face a major challenge: they cannot track mental health changes quickly and respond accordingly (11). Therefore, at present, some scholars are analyzing a large number of social App data and using natural language processing (NLP) methods to explore the emotion of tweet publishers from text information (12). Through the analysis of emotional information, we can obtain the indicators that can respond to the residents' mental health in a timely manner. These indicators range from 0 to 1. The closer is the value to 1, the stronger is the optimism, and the closer is the value to 0, the stronger is the pessimism.

In this article, we chose to use XGBoost to explore the impact of COVID-19 on the mental health of Chinese residents. To obtain real-time text information, we chose to use the text information on social media Sina Weibo for analysis, which is the largest tweet platform in China. The data contain a total of 13 million blogs in China with geographical location tags. The data start date is 1 January 2020 and the end date is 1 March 2020. Furthermore, the detailed data description will be shown in the article. To make rational use of these data, XGBoost is used to analyze and process the data. We use XGBoost because of its strong interpretability and its suitability for exploring sparse data such as text data. These advantages come from its tree model structure. XGBoost can also provide us with the feature importance of the tree model, which we will analyze to check its rationality.

## 2. Related work

Several studies have been conducted to investigate the impact of the COVID-19 pandemic on mental health and wellbeing. Li et al. (13), based on China's microblog platform, surveyed 17,865 active users and analyzed the data from 13 January 2020 to 26 January 2020, using the online ecological recognition model to obtain emotional indicators (e.g., anxiety, depression, identification, and Oxford happiness) and cognitive indicators (e.g., social risk judgment and life satisfaction). Zhang et al. (14) designed cross-sectional data using mobile phone App data and telephone interviews, and studied 263 individuals. Liu et al. (15) designed an online questionnaire to survey 285 residents in Wuhan, China, and designed a PTSD checklist for DSM-5 (PCL-5) from the Pittsburgh Sleep Quality Index (PSQI) to study the questions. Qiu et al. also designed a network questionnaire to study 52,730 individuals and designed the COVID-19 Peritraumatic Distress Index (CPDI) to conduct problem research (16). Wang et al. designed a network questionnaire to study 1,210 individuals and designed the Impact of Event Scale Revised (IES-R) and Depression, Anxiety and Stress Scale 21 (DASS-21) (5).

Moreover, studies have been conducted on medical workers to explore the job burnout and mental health status of medical staff during the pandemic. Lai et al., Kang et al., and Huang et al. (17–19) conducted research on different numbers of medical personnel and used patient health indicators, such as PHQ-9, GAD-7, and ISI-7, to conduct problem research.

## 3. Research design

### 3.1. Social data

In previous studies, some scholars used social media data to predict influenza activity (20) and outbreak (21). In the same way, we decided to use social media data as the data source for our experiments and evaluate the mental health status of Chinese people by these data.

**Figure 1:**
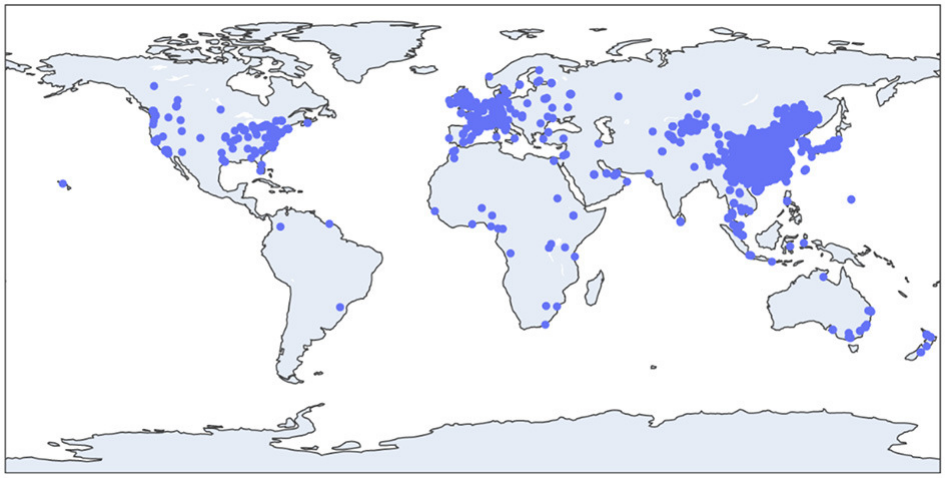
Distribution of location information of tweets on 4 April 2020.

Sina Weibo is the largest blogging platform in China, and millions of users are active on this platform every day (https://www.weibo.com/). Because of the huge and open nature of the platform, many Chinese people publicly publish their living conditions and inner thoughts on Sina Weibo. However, due to the design of the platform, it is very difficult for us to crawl data. Hu et al. previously collected data from Sina Weibo. The data period is from 00:00 (GMT + 8) on 1 December 2019 to 23:59 (GMT + 8) on 30 April 2020 (22). The data contain 33,519,644 pieces of data in total, of which 895,012 are geotagged data. Figure 1 shows the general distribution of the data [Figure 1 is taken from Tian et al. (22)]. See Section 4.1 for a detailed description of data.

### 3.2. Models

Using these social media data, we first use the sentiment analysis tool Sentiment Knowledge Enhanced Pre-training (SKEP) model provided by Baidu for analysis, which was released in 2020. It is an open-source python library and some studies had used it for mental research (22). For each tweet, SKEP can use the input text information as some prior knowledge of sentiment to return two probability values reflecting positive and negative emotions, and the sum of the two probability values is 1. In this article, we use the probability of positive emotions as the expression of the user's mental health status corresponding to this blog.

Because the data contain geographic location tags, and for a certain time end, people in the same city should receive roughly the same anti-epidemic policy, so we use the blog information with geographic location tags of the same city in the same time period for analysis. We used XGBoost to analyze the impact of COVID-19 on the mental health of Chinese people. XGBoost is one of the boosting algorithms. The idea of formulating the boosting algorithm is to integrate many weak classifiers to form a strong classifier. Because XGBoost is a lifting tree model, it integrates many tree models to form a strong classifier. The tree model used is the Classification and Regression Tree model (CART). XGBoost is improved on the basis of GBDT to make it more powerful and applicable to a wider range.

For analysis using XGBoost, we collected the number of COVID-19 infection cases in each city during the corresponding period of social data and constructed the dummy variable feature *COVID*_19_*it* , indicating whether the epidemic occurred in city i at time t, where 1 indicates occurrence and 0 indicates no occurrence. In addition, we constructed the epidemic number variable:


$$num\_COVID\_19=\{\begin{array}{c} number\;of\;infected\;persons,\;if\;COVID_{19_{it}}\text{​}=\text{​}1\\ 0\;if\;COVID_{19_{it}}=0 \end{array}.$$

In addition, we use Jieba word segmentation (https://pypi.org/project/jieba/) to gather statistics on Sina Weibo to form a word vector matrix expressing people's mental health and use sklearn's CountVectorize (https://scikit-learn.org/stable/modules/generated/sklearn.feature_extraction.text.CountVectorizer.html) to gather statistics on the obtained word vectors and select the top 500 words that appear most frequently in the training set to form a word vector matrix. The positive emotion probability obtained by SKEP is used as a tag to explore the impact of social media data on users' mental health.

## 4. Experiments and analysis

### 4.1. Data collection and description

As mentioned in Section 3.1, we use that data set for the experiment. The data start time is from 00:00 (GMT + 8) on 1 December 2019 to 23:59 (GMT + 8) on 30 April 2020, and a total of 895,012 geotagged tweets are included. The information description of each tweet is shown in Table 1. Furthermore, Table 2 shows a specific example of our experiment data.

**Table 1:** Data description.

**Name**	**Description**
_id	The unique identifier of the tweet
User_id	The unique identifier of the user who posted the tweet
Crawl_time	Crawling time of the tweet
Created_at	Creating time of tweet
Like num	The number of like at crawling time
Repost_num	The number of retweet at crawling time
Comment_num	The number of comment at crawling time
Content	The content of tweet
Origin_weibo	The _id of the origin tweet, only not empty when the tweet is a retweet one
Location_map_info	Information of latitude and longitude, only not empty when the tweet contains the location information

**Table 2:** Specific example of experiment data.

**_id**	**Jwm2cyhQQ**
User_id	e0470a66f95fe66d1607931932
Crawl_time	2020-12-01
Created_at	00:00
Like num	0
Repost_num	0
Comment_num	0
Content	[On the way to fight against the epidemic, the scientific expert behind the scenes has gone] He always wanted to go faster as he fought for every second in the vaccine campaign! Due to continuous work and overwork, Professor Zhao Zhendong, a researcher at the Institute of Pathogen Biology of the Chinese Academy of Medical Sciences, died in Beijing on September 17 at the age of 53 after a business trip. Professor Zhao Zhendong was a well-known expert engaged in pathogen biology and infection immunology research in China, and at the beginning of the epidemic, he said, “This... Full story forwarded for reason:[tear]”
Origin_weibo	Jwl894jgH
Location_map_info	None

**Figure 2:**
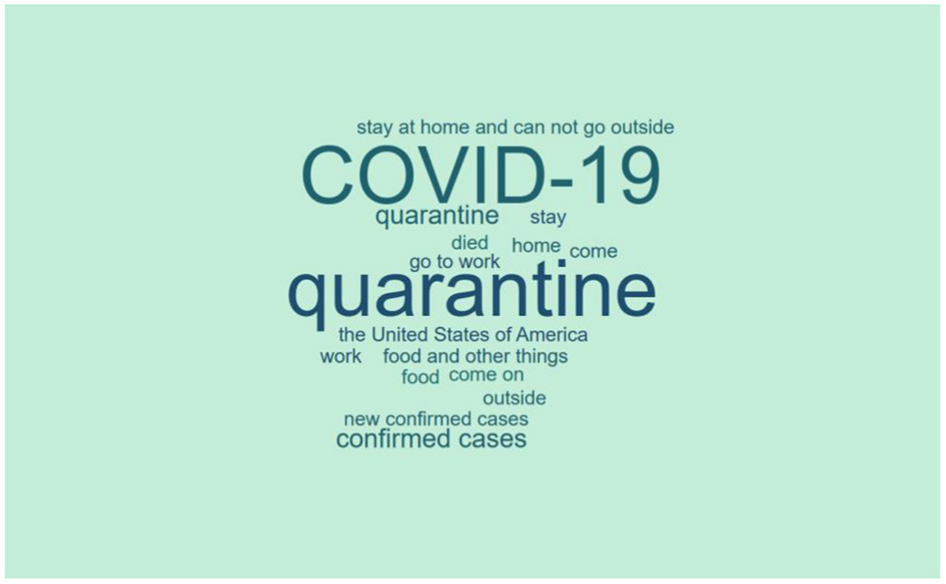
Word cloud of tweets.

In addition, we need to obtain the word frequency matrix of each tweet in the training set. The specific method has been given in Section 3. To have a general understanding of the data, we use Jieba to draw the word cloud. The drawing results are shown in Figure 2. It can be seen that the obtained word cloud reflects the mental health of the user who responded to this tweet to a certain extent.

After using the Jieba word segmentation (only the words corresponding to common nouns, proper nouns, verbs, adverbs, gerund, adjectives, and adverbs are retained as the cleaned data), we also use CountVectorize to carry out word vector statistics and obtain the words with the highest frequency as features. The results are shown in Table 3.

**Table 3:** Results of words with the highest frequency as features.

	**COVID-19**	**Quarantine**	**Confirmed cases**	**Stay at home and can not go outside**	**Come on**	**Food and other things**	**New confirmed cases**	**Died**	**Go to work**	**The United States of America**
1	0	1	0	1	0	1	0	0	0	0
2	0	0	1	0	0	0	1	0	0	0
3	0	0	0	1	1	1	0	0	0	1
4	1	0	0	1	0	0	0	0	0	0
5	0	0	0	0	1	0	0	0	0	0
6	1	0	0	0	0	0	1	0	0	0

### 4.2. Hyperparameters and metrics

Metrics: MSE statistical parameters are the mean value of the square sum of the errors of the corresponding points of the predicted data and the original data. The calculation method is given as follows:


$$\begin{array}{l} MSE=\frac{1}{n}\overset{n}{\underset{i=1}{\sum }}w_{i}{\left(y_{i}-\hat{y_{i}}\right)}^{2} \end{array}$$

In this article, model training is carried out in the form of rolling. The training and testing processes are divided into the following steps:

1. Dataset division: each round of rolling determines the data set within the sample and the data set outside the sample, and divides them in chronological order.
2. Feature and label generation: the text in the sample is vectorized, feature X generated and labeled y, and the words used recorded.
3. Training: *k*-fold cross-training is conducted within the sample, and GridSearchCV of sklearn (sklearn.model_selection.GridSearchCV—scikit-learn 1.1.2 documentation) is used to find the optimal parameters.
4. Out-of-sample preprocessing: word vector on the text outside the sample is used to perform out-of-sample preprocessing, based on the words used in the sample.
5. Prediction and factor construction: the optimal model obtained by cross-validation is used to predict the outside of the sample, and the factor value describing the user's mental health is obtained.

Extreme gradient boosting (XGBoost) is a Boosting integration algorithm that is a strong learner that combines multiple weak learners (such as decision trees) in a series manner, in a way that continuously reduces the loss function by iterating between weak learners. We perform a mesh search of all the hyperparameter combinations of the XGBoost classifier and use five-fold cross-validation to select the lowest set of hyperparameters in the validation set, average loss function as the final hyperparameter of the model, and the hyperparameter settings are shown in Table 4.

**Table 4:** XGBoost hyperparameters' selection.

**Model**	**Hyperparameters**	**Range**
XGBoost	Learning rate	[0.025, 0.05, 0.075]
	Max depth	[3,5]
	Subsample	[0.8, 0.85, 0.9, 0.95]

### 4.3. Results

#### 4.3.1. The impact of COVID-19 epidemic on mental health

**Figure 3:**
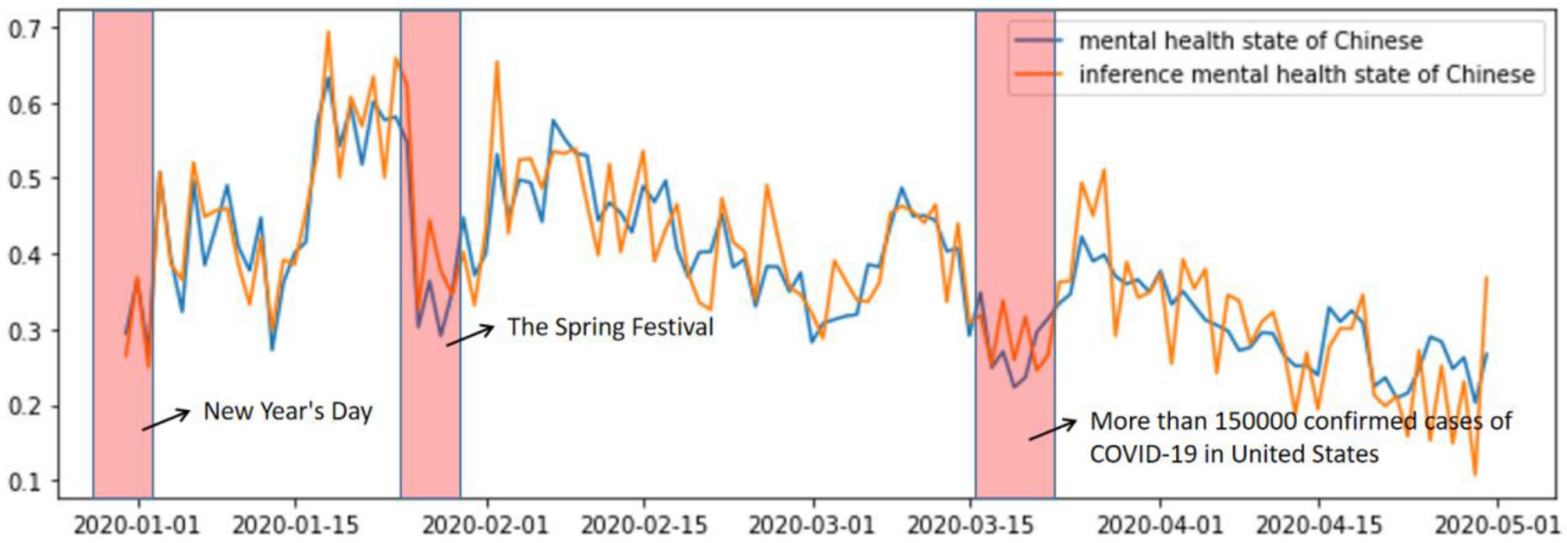
Analysis of inference mental health state.

Figure 3 shows the user's mental health state inferred from the text information by XGBoost and the real user's mental health state obtained by SKEP. From the figure, we can easily conclude that:

1. The COVID-19 pandemic has a significant negative impact on people's mental health, as shown by the clear downward trend in both the real and inferred mental health states over time.
2. The red-striped data in Figure 3 can be divided into two categories. First, Chinese traditional festivals show a significant decrease in users' mental health status, which may be due to the inability to visit relatives and friends during the pandemic. Second, when the number of people diagnosed with COVID-19 in the United States exceeded 150,000 in March, the users' mental health status also declined significantly. This observation may be due to the globalization of the pandemic and its impact on prevention and control measures.

Our explanation for this is given as follows: with the deepening of globalization, every resident on earth understands that the epidemic situation in other countries will have a significant impact on their own prevention and control measures. Therefore, the mental health status of residents also dropped sharply after the news emerged.

#### 4.3.2. Importance influence of words

**Figure 4:**
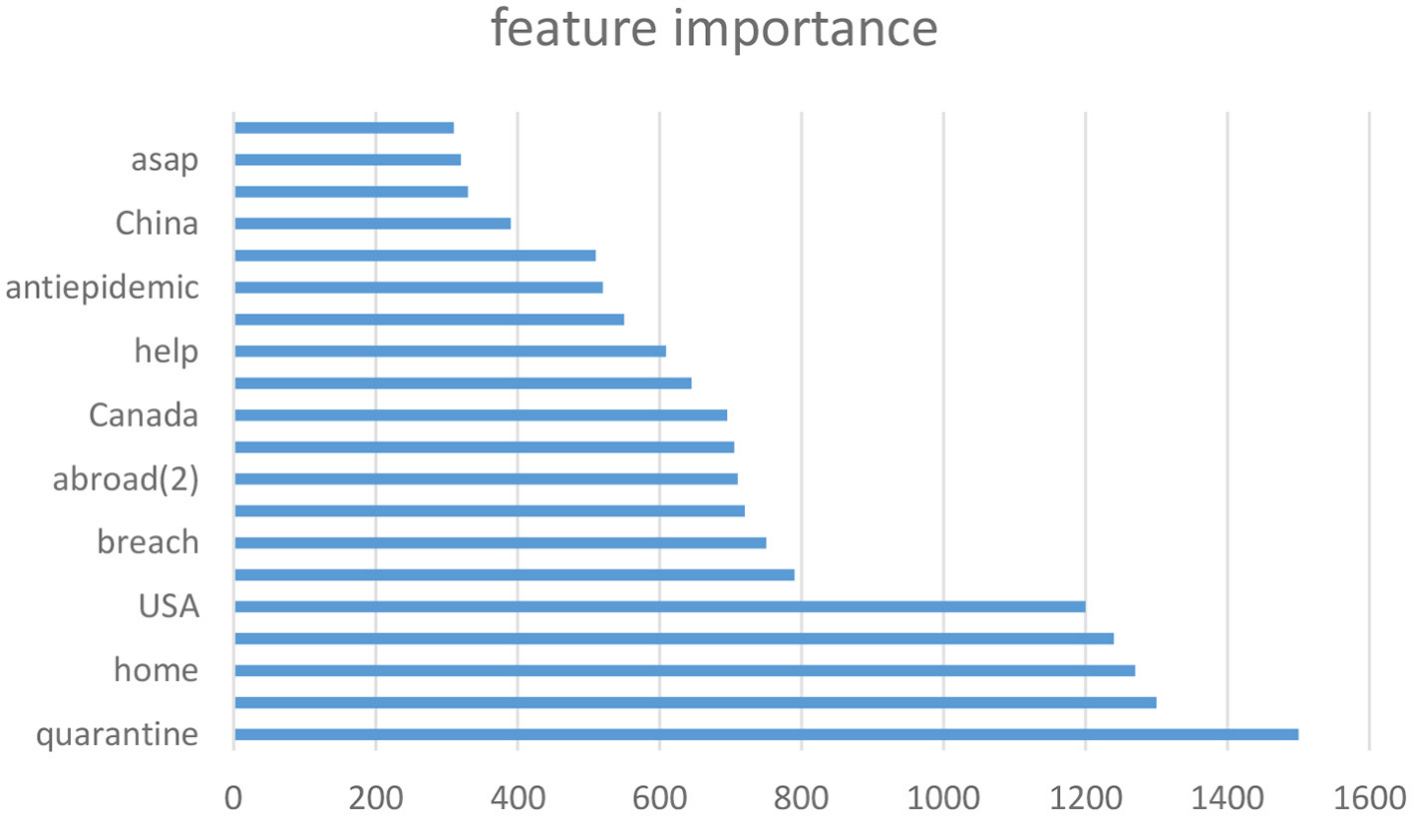
Feature importance.

In Figure 4, the importance of certain characteristics is shown, with “stay at home and can not go outside,” “confirmed cases, and “quarantine” being the top three important features. This fact reflects the significant impact of the policy of “home isolation and no going out” on the mental health of Chinese people, as well as on the concern over the number of confirmed cases. Other words, such as “food,” “Canada,” “help,” and “Spring Festival,” are also representative of the living conditions in China during that time and can accurately reflect the mental health status of the Chinese people.

## 5. Discussion

This study aimed to quantify the impact of the COVID-19 pandemic on people's mental health using social media data on Sina Weibo. By applying the natural language processing technology and a state-of-the-art deep learning framework (SKEP), as well as using the powerful XGBoost machine learning algorithm, we were able to analyze the results and provide empirical evidence of the impact of the pandemic on people's mental health.

Our findings indicate that the COVID-19 pandemic has had a significant negative impact on the mental health of people. This is consistent with a previous research on the effects of pandemic on mental health. We call on the general public to care more for people around us and work together to overcome the challenges presented by the pandemic.

We also found that Chinese traditional festivals are important for maintaining relationships between relatives and friends, and that the inability to visit loved ones during the pandemic had a significant impact on people's mental health. We recommend that policymakers take this into account when planning public health measures and suggest finding alternative ways for people to connect with each other during these festivals.

Overall, our study provides important implications for both the field of natural language processing and the field of public health, demonstrating how social text data can be used to measure and analyze the mental health of users during the pandemic, and how our findings can help public health policymakers to understand and improve the psychological wellbeing of the population.

## 6. Conclusion and suggestions

### 6.1. Conclusion

In this article, our analysis of social media data from Sina Weibo shows that the COVID-19 pandemic has a significant negative impact on people's mental health. After using the related natural language processing technology, XGBoost is used to analyze the results. The empirical results show that COVID-19 has a significant impact on people's mental health. Therefore, we call on the general public to care more for people around us and to let us tide over the difficulties together. In addition, we also found that Chinese traditional festivals are important festivals for maintaining relations between relatives and friends. Due to the pandemic, people cannot visit relatives and friends, and it is difficult for them to accept this truth. Therefore, their psychological state drops sharply. With the deepening of globalization, every resident on earth understands that the epidemic situation in other countries will have a significant impact on their own prevention and control measures. Therefore, the serious news of the epidemic situation in foreign countries will also have an impact on the mental health of residents.

### 6.2. Strengths and limitations

This article has several strengths that contribute to the literature on the impact of COVID-19 on mental health. First, it uses a large and rich data source of social text data from Sina Weibo, which can capture the real-time and diverse opinions and emotions of users during the COVID-19 pandemic. Second, it applies a state-of-the-art deep learning framework (SKEP) to measure the user's mental state based on the sentiment knowledge, which can provide more accurate and fine-grained results than traditional methods. Third, it employs a powerful machine learning algorithm (XGBoost) to study the relationship between the word vector sparse matrix and the mental health state of the users, which can handle high-dimensional and sparse data efficiently and effectively.

However, this article also has some limitations that need to be addressed in a future study. First, it only focuses on one platform (Sina Weibo) and one country (China), which may limit the generalizability and applicability of the findings to other contexts and populations. Future study should include more platforms and countries to compare and contrast the results. Second, it does not consider other factors that may affect the user's mental health, such as demographic variables, social support, and coping strategies, which may confound or moderate the impact of COVID-19. Future study should take steps to control these factors or explore their interactions with COVID-19. Third, it uses a single indicator (the probability of positive or negative mental state) to measure the user's mental health, which may not capture the complexity and diversity of mental health issues. Future study should use more professional and comprehensive mental health indicators, such as depression scales, anxiety scales, or stress scales.

### 6.3. Future vision

For future study, we plan to improve our research in three aspects. First, we aim to use more complete data sets that include not only Weibo, but also other platforms, such as WeChat, TikTok, and Kwai, where news messages are presented in text or short video form. This development will help us to further explore the impact of COVID-19 on mental health from different perspectives. Second, we intend to design our own model that can capture time and space information, such as the user's experience of isolation control and the user's location. We can use graph neural network to study the problem by considering the user information of the entire city. Third, we hope to use more professional mental health indicators for measuring the user's mental state, which will provide more value to study the problem. By doing so, we expect to have important implications for both the field of natural language processing and the field of public health, as our article demonstrates how social text data can be used to measure and analyze the mental health of users during the pandemic, and how our findings can help public health policymakers to understand and improve the psychological wellbeing of the population.

## Data availability statement

The original contributions presented in the study are included in the article/supplementary material, further inquiries can be directed to the corresponding author.

## Author contributions

All authors listed have made a substantial, direct, and intellectual contribution to the work and approved it for publication.

## References

[1] World Health Organization. Coronavirus Disease (COVID-19) Pandemic. (2020). Available onlline at: https://www.who.int/europe/emergencies/situations/covid-19 (accessed February 01, 2023).

[2] WHO. Statement on the Second Meeting of the International Health Regulations (2005). Emergency Committee Regarding the Outbreak of Novel Coronavirus (2019-nCoV). Geneva: World Health Organization (2020).

[3] Holmes EA O'Connor RC Perry VH Tracey I Wessely S Arseneault L. Multidisciplinary research priorities for the COVID-19 pandemic: a call for action for mental health science. Lancet Psychiatry. (2020) 7:547–60. 10.1016/S2215-0366(20)30168-1 32304649 PMC7159850

[4] Liu N Zhang F Wei C Jia Y Shang Z Sun L. Prevalence and predictors of PTSS during COVID-19 outbreak in China hardest-hit areas: gender differences matter. Psychiatry Res. (2020) 287:112921. 10.1016/j.psychres.2020.112921 32240896 PMC7102622

[5] Wang C Pan R Wan X Tan Y Xu L Ho CS. Immediate psychological responses and associated factors during the initial stage of the 2019 coronavirus disease (COVID-19) epidemic among the general population in China. Int J Environ Res Public Health. (2020) 17:1729. 10.3390/ijerph17051729 32155789 PMC7084952

[6] World Health Organization. Policy Brief: COVID-19 and the Need for Action on Mental Health. (2020). Available online at: https://www.who.int/publications/i/item/9789240015111 (accessed February 07, 2023).

[7] Statista Research Department. Total Number of Suicides Committed in Japan from 2012 to 2021. Hamburg (2022), p. 1.

[8] Ministry of Health. COVID-19 Mental Wellness Taskforce Report. (2020). Available online at: https://www.moh.gov.sg/resources-statistics/reports/covid-19-mental-wellness-taskforce-report (accessed February 08, 2023).

[9] Gao J Zheng P Jia Y Chen H Mao Y Chen S. Mental health problems and social media exposure during COVID-19 outbreak. PLoS ONE. (2022) 15:e0231924. 10.1371/journal.pone.0231924 32298385 PMC7162477

[10] Hao F Tan W Jiang L Zhang L Zhao X Zou Y. Do psychiatric patients experience more psychiatric symptoms during COVID-19 pandemic and lockdown? A case-control study with service and research implications for immunopsychiatry. Brain Behav Immunity. (2020) 87:100–6. 10.1016/j.bbi.2020.04.069 32353518 PMC7184991

[11] Areán PA Ly KH Andersson G. Mobile technology for mental health assessment. Dialogues Clin Neurosci. (2016) 18:163–9. 10.31887/DCNS.2016.18.2/parean 27489456 PMC4969703

[12] Conway M Daniel O. Social media, big data, and mental health: current advances and ethical implications. Curr Opin Psychol. (2016) 9:77–82. 10.1016/j.copsyc.2016.01.004 27042689 PMC4815031

[13] Li S Zhang Y. Mental healthcare for psychiatric inpatients during the COVID-19 epidemic. Gen Psych. (2020) 33:e100216. 10.1136/gpsych-2020-100216 32363326 PMC7174023

[14] Zhang Y Ma ZF. Impact of the COVID-19 pandemic on mental health and quality of life among local residents in Liaoning Province, China: a cross-sectional study. Int J Environ Res Public Health. (2020) 17:2381. 10.3390/ijerph17072381 32244498 PMC7177660

[15] Liu S Yang L Zhang C Xiang Y-T Liu Z Hu S. Online mental health services in China during the COVID-19 outbreak. Lancet Psychiatry. (2020) 7:e17–8. 10.1016/S2215-0366(20)30077-8 32085841 PMC7129099

[16] Qiu J Shen B Zhao M Wang Z Xie B Xu Y. A nationwide survey of psychological distress among Chinese people in the COVID-19 epidemic: implications and policy recommendations. Gen Psych. (2020). 33:e100213. 10.1136/gpsych-2020-100213 32215365 PMC7061893

[17] Lai J Ma S Wang Y Cai Z Hu J Wei N. Factors associated with mental health outcomes among health care workers exposed to coronavirus disease 2019. JAMA Netw Open. (2020) 3:e203976. 10.1001/jamanetworkopen.2020.3976 32202646 PMC7090843

[18] Kang L Ma S Chen M Yang J Wang Y Li R. Impact on mental health and perceptions of psychological care among medical and nursing staff in Wuhan during the 2019 novel coronavirus disease outbreak: a cross-sectional study. Brain Behav Immun. (2020) 87:11–7. 10.1016/j.bbi.2020.03.028 32240764 PMC7118532

[19] Huang JZ Han MF Luo TD Ren AK Zhou XP. Mental health survey of medical staff in a tertiary infectious disease hospital for COVID-19. Zhonghua Lao Dong Wei Sheng Zhi Ye Bing Za Zhi. (2020) 20:192–5. 10.3760/cma.j.cn121094-20200219-00063 32131151

[20] Zhang J Centola D. Social networks and health: new developments in diffusion, online and offline. Annu Rev Sociol. (2019) 45:91–109. 10.1146/annurev-soc-073117-041421

[21] Ginsberg J Mohebbi MH Patel RS Brammer L Smolinski MS Brilliant L. Detecting influenza epidemics using search engine query data. Nature. (2009) 457:1012–4. 10.1038/nature07634 19020500

[22] Tian H Gao C Xiao X Liu H He B Wu H. SKEP: sentiment knowledge enhanced pre-training for sentiment analysis. arXiv. (2005). [preprint]. 10.48550/arXiv.2005.05635

